# Prevalence and Risk Factors of Maternal Anemia Among Pregnant Women in Central Jordan

**DOI:** 10.1155/anem/5554286

**Published:** 2026-09-30

**Authors:** Noor Abu Eid, Omar Abueed, Maya Abuarayes, Reham Khresheh

**Affiliations:** ^1^ Faculty of Nursing Mutah University Mu’tah Jordan mutah.edu.jo; ^2^ School of Systems Science and Industrial Engineering Binghamton University Binghamton New York USA binghamton.edu

**Keywords:** antenatal care, iron deficiency, maternal anemia, maternal health, pregnancy outcomes, public health interventions, risk factors

## Abstract

Maternal anemia remains one of the most significant public health issues, particularly in low‐to‐middle‐income countries. The World Health Organization reported a maternal anemia prevalence of 37% among women of reproductive age; this rate is further inflated in developing countries. This cross‐sectional analytical study aimed to estimate the prevalence of maternal anemia and assess its associated risk factors among pregnant women receiving maternity services in central Jordan. Data collection was conducted using validated self‐administered questionnaires administered to 297 pregnant women receiving medical care at three medical facilities during the period between August and November of 2023. The questionnaire examined several domains related to maternal anemia, including sociodemographic characteristics, pregnancy profile, reproductive history, nutritional habits, hemoglobin levels, and evaluated the knowledge level of anemia among pregnant women. Statistical analyses were conducted using the Statistical Package for the Social Sciences (SPSS), with multivariable logistic regression as the primary inferential model, supplemented by a multilayer perceptron neural network to validate predictor consistency. The models achieved an accuracy of 73.0% and 71.1%, respectively. The results revealed that approximately half of the participants (52.5%) were anemic. A multivariable logistic regression was used to identify the associated risk factors for maternal anemia. Anemia is more likely to develop in later stages of pregnancy (odds ratio [OR] = 1.69, 95% confidence interval [CI] 1.036–2.771) and among women with a high number of abortions (OR = 6.33, 95% [CI] 1.314–13.515), whereas the use of nutritional supplements was a protective factor (OR = 0.104, 95% [CI] 0.013–0.858). These results highlight the urgent need for strengthened antenatal screening protocols and targeted nutritional interventions, particularly for women in later stages of pregnancy and those with a history of abortions.

## 1. Introduction

Maternal anemia is still a major public health problem worldwide, and especially in developing countries, where it contributes to poor health outcomes for both mothers and neonates. The World Health Organization (WHO) defines anemia in pregnancy as a hemoglobin (Hb) level below 11.0 g/dL and grades it as mild, moderate, or severe depending on the extent of the decline in Hb levels [1, 2].

Global nutrition and maternal health initiatives have made modest progress regarding anemia prevalence among women of reproductive age between 2000 and 2018 [3]. Pregnancy increases risk through natural hemodilution, increased iron requirements, and higher metabolic demands, especially in later gestation. These demands are partly met based on pregnant women’s nutrition, reproductive history, and the consistent use of supplements. Maternal anemia can lead to serious health problems, including postpartum hemorrhage, preterm birth, low birth weight, poor fetal growth, and even maternal death. Therefore, early detection and prevention within antenatal care systems are essential [4].

Anemia during pregnancy varies widely across regions, shaped by differences in diet, reproductive history, and access to antenatal care. This reality suggests that local assessment matters more than regional average rates. In the Gulf region, maternal anemia prevalence was reported at 34.1% in northern Saudi Arabia and 35.4% in Qatar. The main associated factors were income, family size, parity (number of births), longer menstrual cycles, gestational age, and interpregnancy interval [5, 6].

In Egypt, Eweis et al. [7] reported an even higher prevalence of 72% among participants at Beni‐Suef University Hospital. The prevalence was tied to poor dietary lifestyle and irregular iron supplement use. Generally, studies across Africa and the Middle East consistently report moderate to high anemia in pregnancy, driven by gestational age, number of children, and poor dietary behaviors [8, 9].

In Jordan, recent national nutrition surveys show that anemia among women of reproductive age has risen over time, reaching 43% in 2017–2018 (up from 26% in 2002) [10]. The prevalence also varies by population and setting within the country. Arabyat et al. [11] reported 37.3% anemia among ever‐married women, with higher odds in urban populations. Abdo et al. [12] found that the prevalence of anemia was 27.4% in pregnant and 19.3% in nonpregnant women, with mild anemia the most common form, and rates vary by gender, age, region, marital status, and education. In southern Jordan, Al‐Kharabsheh et al. [13] found the prevalence rate at 36.8% and concluded that Hb levels are tied to education, income, diet, antenatal care regularity, and supplement use. Whereas Awidi et al. [14] concluded that severe anemia was strongly tied to heavy menstrual bleeding and abortion history. Surveys conducted in Jordan show that maternal anemia is still a real problem despite several maternal health programs. Earlier work has tied anemia to obstetric factors like parity, stage of pregnancy, past reproductive outcomes, and delivery‐related risks [15–17].

Limited awareness of maternal anemia, its causes, and symptoms may lead to a higher prevalence rate [18]. Intervention studies show that structured health education improves anemia knowledge, dietary behavior, and supplement adherence [19, 20]. However, the work of Tayyem et al. [21] found that supplement use was widespread, especially in later pregnancy, but often started late or was not tailored to clinical need. Facility‐level evidence remains limited, leaving a comprehensive characterization of the magnitude, severity distribution, and locally modifiable determinants underexplored. In addition, the current research examined prevalence, knowledge, nutrition, and obstetric factors separately, rather than within a single integrated analysis.

Most Jordanian studies have also relied on descriptive methods and conventional regression, leaving little room for predictive or data‐driven models that could flag higher‐risk groups. As a result, few studies have brought the key pieces together by estimating prevalence while examining risk factors, nutritional behaviors, and maternal knowledge in a unified analysis that uses both conventional regression and machine learning. This gap makes it harder to design evidence‐based antenatal screening, optimize resource allocation, and develop targeted interventions. The present study addresses it with a context‐specific assessment of maternal anemia among pregnant women attending antenatal care in central Jordan, estimating its prevalence and identifying its main predictors using both multivariable logistic regression and machine learning.

The remainder of this study is structured as follows: Section 2 explains the study design, setting, participants, data collection instrument, and statistical analysis procedures. In Section 3, we present the study results as prevalence estimates obtained using multivariable logistic regression models and a multilayer perceptron (MLP) network. Section 4 discusses the findings and outlines implications for antenatal screening and targeted counseling. Finally, Section 5 outlines study limitations and concludes with clinical and research implications.

## 2. Materials and Methods

### 2.1. Study Design and Population

The participants in this study were pregnant women of reproductive age receiving antenatal care at one of three facilities in Amman, Jordan: Al‐Basher Hospital, Al‐Jubaiha Comprehensive Health Center, and Al‐Hashemi Comprehensive Health Center. These facilities are among the largest government maternal health providers in Amman. Selection was conducted in coordination with the Ministry of Health (MoH) maternal unit, which recommended these sites and facilitated access and data collection. These public facilities provide antenatal care at low cost and serve a diverse population of pregnant women, predominantly from low‐ and middle‐income families in Amman and neighboring governorates of central Jordan. The MoH also indicated that these communities have received limited attention in previous maternal health research. Therefore, the sample is considered a reasonable representation of women utilizing public antenatal care in central Jordan, although those attending private clinics were not included.

Eligibility criteria included women aged 18–45 years, in the second or third trimester, attending routine antenatal visits, able to read and write Arabic, and willing to participate and provide informed consent. Women with complicated pregnancies, specifically those with gestational diabetes, preeclampsia, placenta previa, or multiple gestations, were excluded. These exclusions were implemented to minimize confounding by clinical illness and to allow for a clearer assessment of associations between anemia and modifiable behavioral and nutritional factors.

A cross‐sectional analytical design was used, as it is efficient and well‐suited to estimating prevalence and examining associations at a single time point [22]. This allowed anemia status and its potential determinants to be measured together within the same assessment period. Data were collected from August to November 2023. Participants were recruited by convenience sampling: All eligible pregnant women attending the three facilities during the study period were invited until the target sample size was reached. The sample size was calculated from the facilities’ attendance records using the Cochran formula, assuming an expected anemia prevalence of 37% from a previous Jordanian study, a 95% confidence level, and a 5% margin of error, giving a minimum of 297 participants [23]. The formulas are given in the following equations:

(1) n=N01+N0−1/N.

(2) N0 =Z2∗P∗1−pε2.

where *Z* is the standard score, *ε* is the margin of error, *N* is the population size, *N*_0 _, the sample size, and $\overset{\wedge }{p}$ is the population proportion.

### 2.2. Measurement and Definition of Anemia

Hemoglobin level was measured at each facility during routine antenatal care using automated hematology analyzers, and we recorded these values for every participant. Anemia was defined as an Hb level below 11.0 g/dL. We applied this uniform threshold across both trimesters rather than a lower trimester‐specific cutoff to keep our prevalence estimate comparable with previous regional and Jordanian studies. Severity was graded as mild (9.0–10.9 g/dL), moderate (7.0–8.9 g/dL), or severe (below 7.0 g/dL). Because the Hb came from facility laboratory testing rather than self‐report, the anemia status is based on an objective clinical measurement. The blood test and the questionnaire were completed at the same antenatal visit, so both sets of data refer to the same time point.

### 2.3. Study Instruments

This study used a validated, self‐administered questionnaire adapted from Alflah et al. [24], who had established its validity and reliability in a similar pregnant population in Yemen. It was developed and administered in Arabic. Before data collection, we pretested the instrument with 20 pregnant women to assess clarity and cultural appropriateness, making minor wording adjustments based on their feedback.

The questionnaire captured key domains relevant to maternal anemia. These included (i) sociodemographic data (i.e., age, residence, occupation, education level, and income); (ii) pregnancy profile (i.e., trimester, pregnancy order, live births, abortions, interpregnancy intervals, and chronic diseases); (iii) nutritional habits (i.e., supplement use including iron tablets, folic acid, and vitamin C; dietary intake of meat, chicken, fish, legumes, eggs, vegetables, fruits, and beverage consumption); (iv) laboratory results for Hb level in g/dL; and (v) the assessment of participants’ knowledge of anemia. Before implementation, the questionnaire was reviewed by two faculty members specializing in maternal and child health nursing, who evaluated the instrument for content relevance, clarity, and cultural appropriateness for the study objectives.

Anemia knowledge was assessed with a 13‐item yes/no questionnaire. The item “Have you heard about anemia?” was excluded from scoring, leaving 12 items scored (1 point per correct answer; total 0–12). Knowledge was classified as poor (< 60%; score 0–7), intermediate (60%–80%; score 8–9), or high (> 80%; score 10–12). The selection of assessed variables was informed by the existing regional literature on maternal anemia in the Gulf region, Egypt, and Yemen, which consistently identified obstetric history, gestational age, supplement use, and nutritional behaviors as dominant associated factors in comparable populations.

### 2.4. Data Management and Analysis

The statistical analyses were performed using Statistical Package for the Social Sciences (SPSS) software Version 28. Before moving to inferential statistics, we screened the data for missing values and outliers and set the statistical significance level at 0.05. The chi‐square test examined bivariate associations among categorical variables, such as demographic data, knowledge of anemia, and attitudes toward nutrition. At the same time, point‐biserial correlations assessed the relationship between anemia and continuous variables, such as age and knowledge score. The assumptions of each test were verified; for instance, the point‐biserial correlation required linearity and normality, and binary logistic regression required a binary outcome and no multicollinearity. Variance inflation factors (VIFs) confirmed no multicollinearity, with values remaining low for every predictor: trimester (1.006), number of abortions (1.013), supplement use (1.030), and tea consumption (1.033), all well below the conventional threshold of 5.

Multivariable logistic regression was selected as the primary inferential model because it produces adjusted odds ratios (ORs), which show the strength and direction of each predictor’s association with anemia while assuming a linear relationship between the predictors and the log‐odds of the outcome. That assumption keeps the effect sizes easy to interpret clinically. As a complement, we added a MLP as an exploratory classifier. Unlike logistic regression, the MLP is a feedforward network that can model nonlinear relationships and interactions among predictors through its hidden layer, without the analyst having to specify those relationships in advance.

The MLP was implemented using SPSS Version 28 with the following configuration: four input neurons corresponding to the candidate predictors (gestational trimester, number of abortions, supplement use, and tea consumption), and one hidden layer with three neurons using the sigmoid activation function. The network training used the scaled conjugate gradient algorithm with cross‐entropy as the error function. Given the modest sample size, we applied the default split of 70% training and 30% testing to limit overfitting and let SPSS select the number of hidden neurons automatically. The MLP was not meant to replace logistic regression but to act as a triangulation check: If a linear model (logistic regression) and a nonlinear one (MLP) point to the same influential predictors, confidence in those predictors increases regardless of the functional form. Model performance was evaluated using classification accuracy and receiver operating characteristic (ROC) curve analysis with the area under the curve (AUC).

## 3. Results

### 3.1. Questionnaire Descriptive Analysis

A total of 297 pregnant women were enrolled in the study, with a mean age of 33.6 years (± 6.15). Most of them (83.5%) resided in urban areas, while the employment status was split almost evenly. About 61.6% held a university degree, 8.8% a postgraduate degree, and more than half (58.6%) reported that their income covered their basic needs. In terms of pregnancy history, 56.9% were in their second trimester; 36.0% were in their fourth pregnancy or more, while 13.5% were pregnant for the first time. About 41.8% of the participants had at least one abortion, and spacing between pregnancies also varied among participants, with 35.0% reporting a 2‐year gap and 46.5% indicating a 3‐year gap.

Moreover, the survey showed that supplement use was common among the participants. Most participants (63.6%) regularly took iron tablets and vitamins during pregnancy, and 76.8% regularly took folic acid during the first 3 months of pregnancy; only 3.4% reported never using supplements. Vitamin C, taken alongside these supplements, was less consistent: 37.0% used it only sometimes, and 29.6% never used it. Tea and coffee were popular, with 53.5% always drinking coffee. With respect to dietary habits, 75.4% of participants occasionally consumed red meat, and 62.6% occasionally consumed chicken, while eating fish was relatively infrequent. Citrus fruits were sometimes too often eaten by 58.6%, and fresh fruits and vegetables were eaten occasionally (46.1% and 48.8%, respectively).

### 3.2. Maternal Anemia Knowledge Assessment

Pregnant women’s awareness of anemia was assessed using a 13‐item questionnaire with binary (yes/no) responses. Most (88.2%) were aware of anemia, and most of that group correctly knew it was associated with food (92.4%). Over 60% of participants recognized anemia risk as a threat to their health and the possibility of accumulating the risk for anemia in women with multiple pregnancies. In addition, 64.1% reported that avoiding tea after meals can reduce anemia risk, whereas 46.6% and 49.2% linked low meat intake and abortion history, respectively, to increased anemia risk. Most pregnant women correctly identified the clinical symptoms of anemia. Approximately 86.0% recognized dizziness and disturbed sleep as common signs of anemia, and 71.0% identified headache and difficulty concentrating. The mean knowledge score was 8.35 ± 2.71 out of 12. Overall knowledge level was moderate to high in most participants, with 42.7% classified as high, 24.4% as intermediate, and 32.8% as poor.

### 3.3. Prevalence, Association, and Significant Factors

Table 1 presents the prevalence of anemia among participants. The average Hb was 11.03 ± 1.66 g/dL (range: 5–15). In total, 52.5% of the participants were anemic (*n* = 156), and 47.5% were non‐anemic (*n* = 141). Most anemic women were mildly anemic (84.6%, *n* = 132), followed by moderately anemic (9.6%, *n* = 15) and severely anemic (5.8%, *n* = 9).

**TABLE 1:** Prevalence and severity distribution of maternal anemia among participants.

	Mean	Std	Min–max	Anemia prevalence		^∗^Anemia *n* = 156	
Hemoglobin level (Hb) g/dl	11.03	1.66	5–15	Anemic	156 (52.5%)	Mild	132 (84.6%)
				Non‐anemic	141 (47.5%)	Moderate	15 (9.6%)
						Severe	9 (5.8%)

^∗^9.0–10.9 g/dL mild anemia, 7.0–8.9 g/dL moderate anemia, below 7.0 g/dL severe anemia.

Table 2 summarizes factors significantly associated with maternal anemia. The results showed that the prevalence of anemia was higher in the third trimester (59.4%) than in the second trimester (47.3%) (*p* = 0.040). A moderately strong positive gradient was found with a history of abortion in the anemia prevalence of 85.7% among those who had reported ≥ 3 abortions (*p* = 0.029). Supplement use was also clinically relevant; 90.0% of women who did not use nutritional supplements had anemia (*p* = 0.020). Lastly, those who reported regular tea consumption had a higher prevalence of anemia (60.4%; *p* = 0.048).

**TABLE 2:** The significance of the association between the key risk factors and maternal anemia (*N* = 297).

Factors	Categories	Non‐anemic	Anemic	*X* ^2^	*p* value
		*N* (%)	*N* (%)		
Pregnancy period	The 2^nd^ trimester	89 (52.7%)	80 (47.3%)	4.233	0.040
	The 3^rd^ trimester	52 (40.6%)	76 (59.4%)		

Number of abortions	None	91 (52.6%)	82 (47.4%)	8.998	0.029
	One	35 (45.5%)	42 (54.5%)		
	Two	13 (39.4%)	20 (60.6%)		
	Third or more	2 (14.3%)	12 (85.7%)		

The use of nutritional supplements	I do not use it	1 (10.0%)	9 (90.0%)	9.847	0.020
	Rare	7 (31.8%)	15 (68.2%)		
	Sometimes	34 (44.7%)	42 (55.3%)		
	Yes, regularly	99 (52.4%)	90 (47.6%)		

Tea consumption	Rarely	44 (44.9%)	54 (55.1%)	6.060	0.048
	Sometimes	61 (56.5%)	47 (43.5%)		
	Always	36 (39.6%)	55 (60.4%)		

### 3.4. Predictive Modeling for Anemia Risk Assessment

The analysis identified multiple characteristics associated with maternal anemia. Candidate predictors identified from the bivariate analysis (e.g., gestational trimester, abortion history, and supplement use) were evaluated in multivariable modeling to identify independent predictors of anemia. The aforementioned factors were integrated into a multivariable logistic regression model employing the backward elimination method. The OR ratios revealed that pregnancy trimester, number of abortions, and the use of supplements jointly accounted for 12.3% of the variance in anemia. We evaluated each risk variable using ORs. Comparison of women in the third versus second trimester revealed a 1.7 times increased risk for developing anemia (OR = 1.69, 95% confidence interval [CI]: 1.036–2.771). Furthermore, women who experienced three or more abortions had a 6.33‐times increased likelihood of developing anemia compared to those with no abortion history (OR = 6.33, 95% CI: 1.314–13.515).

By contrast, regular use of nutritional supplements (iron tablets and vitamins) was protective: Women who used them consistently had an OR of 0.104, an 89.6% reduction in the odds of anemia (1 − OR). Tea consumption contributed little and was dropped from the final model. Overall, the model classified anemia with 73% accuracy, as shown in Table 3. Figure 1 shows the ROC curve for the logistic regression model in maternal anemia classification. The Hosmer–Lemeshow test confirmed adequate model fit (χ^2^ = 7.306, df = 8, *p* = 0.504), showing that predicted probabilities did not differ significantly from observed outcomes across risk groups. The omnibus test of model coefficients confirmed overall statistical significance of the model (χ^2^ = 28.671, df = 9, *p* < 0.001), indicating that the set of predictors collectively improves classification over the null model.

**TABLE 3:** Multivariable logistic regression results for maternal anemia predictors among pregnant women^∗^.

Predictors	B	Wald	*p* value	Exp (B)	95% CI for EXP (B)	
					Lower	Upper
Period of the current pregnancy (third vs. second trimester)	0.527	4.407	0.036	1.69	1.036	2.771
Number of abortions (three or more vs. non)	1.846	5.291	0.021	6.33	1.314	13.515
Nutritional supplements (yes, regularly vs. never used)	−2.267	4.418	0.036	0.104	0.013	0.858

^∗^Nagelkerke *R*‐square 12.3%, accuracy 73.0%.

**FIGURE 1:**
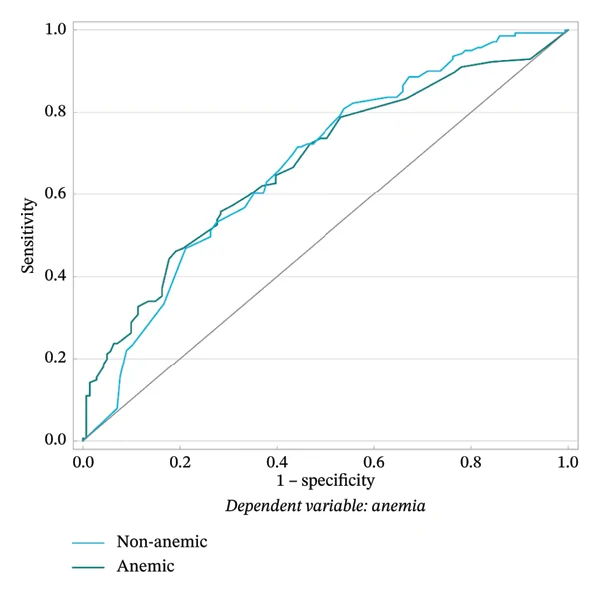
Receiver operating characteristic (ROC) curve for the logistic regression model in maternal anemia classification.

Additionally, a MLP artificial neural network was incorporated as a complementary, data‐driven classifier to capture complex, nonlinear relationships in the data and further assess the finding’s robustness. The goal was not to replace logistic regression inference but to confirm whether the same predictors remained most influential when allowing for nonlinear patterns. Figure 2 shows the MLP architecture, consisting of four input variables and one hidden layer with three neurons, each with a sigmoid activation function. Figure 3 shows the feature importances for the input variables from the best‐performing model. The results of the study reveal that consumption of supplements (such as iron tablets) during pregnancy was the most influential factor, followed by the number of prior abortions and pregnancy trimesters.

**FIGURE 2:**
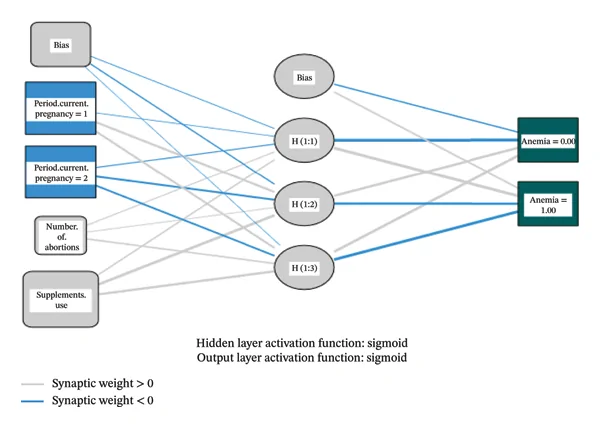
Multilayer perceptron (MLP) neural network structure applied to predict maternal anemia risk.

**FIGURE 3:**
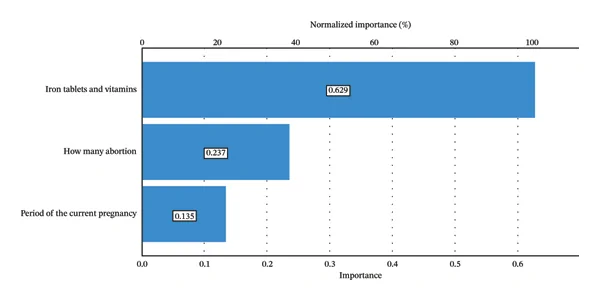
Predictor importance profile from the MLP model, highlighting variables most strongly contributing to anemia classification.

Both models were evaluated by classification accuracy and ROC analysis. The MLP reached an accuracy of 71.1%, and its ROC curve, shown in Figure 4, yielded an AUC of 0.718, indicating a good ability to distinguish anemic from non‐anemic women and supporting its use for anemia classification in this population.

**FIGURE 4:**
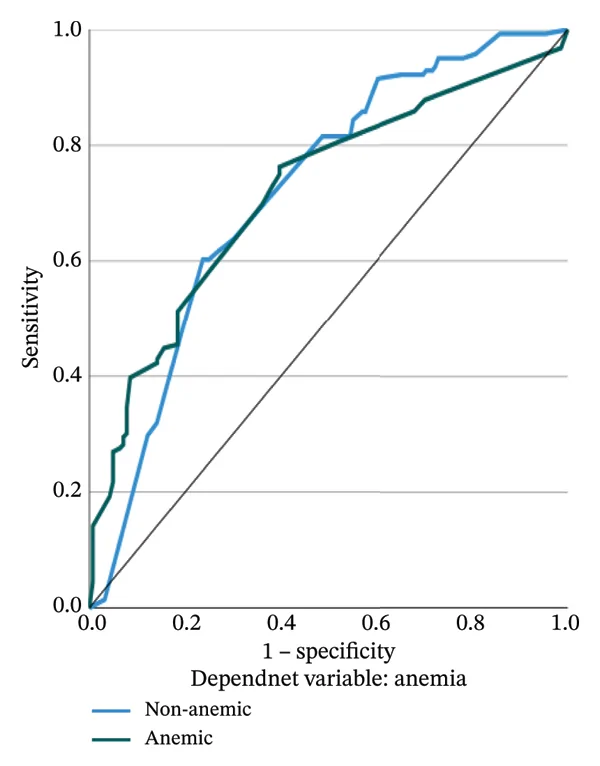
Receiver operating characteristic (ROC) curve for the multilayer perceptron (MLP) model in maternal anemia classification.

The logistic regression model achieved a comparatively higher accuracy of 73.0%, further supporting its suitability as the primary inferential model. Across both the logistic regression and MLP models, similar predictors prove to be consistently informative: regular intake of supplements (protective), multiple abortions (higher risk), and trimester of gestation being later in pregnancy (greater risk). Consistent with the descriptive findings, anemia prevalence in the third trimester was 59.4%, and it reached 85.7% among women with ≥ 3 abortions (Table 2), while women who reported no supplement use had 90.0% anemia prevalence. These findings define clinically informative subgroups for risk stratification in routine antenatal care.

The concordance between the logistic regression and MLP models in predictor ranking and classification accuracy suggests that the relationships between the identified risk factors and maternal anemia in this sample are predominantly linear and additive. The MLP did not reveal any nonlinear patterns or interactions beyond what logistic regression already captured. This is itself a useful result: It suggests that the linearity assumption behind the logistic model holds reasonably well for these data, so its ORs can be read with greater confidence. The MLP was still worth including, since we could not have reached this conclusion without a model capable of detecting nonlinearity.

## 4. Discussion and Implications

The study’s findings estimated a prevalence of maternal anemia at 52.5% in central Jordan. This result indicates a higher prevalence than that reported by other scholars in the region. This prevalence exceeds rates reported in the Gulf region; northern Saudi Arabia was at 34.1%, whereas Qatar was at 35.4%. Maternal anemia was associated mainly with income, family size, parity, and longer menstrual cycles [5]. However, the findings aligned with surveys from Egypt. Eweis et al. [7] reported a higher prevalence of 72%. The main risk factors matched those observed in Jordan, including maternal age and parity, inadequate diet and beverage habits, and inconsistent supplement intake. This suggests that countries with similar socioeconomic and health‐system profiles face comparable anemia burdens, unlike the Gulf states.

Several factors may explain Jordan’s higher prevalence compared to Gulf countries. Nutritional intake may differ based on access to and consumption of iron‐rich foods. Gulf countries also have higher average incomes, which may reduce financial barriers to a more varied, iron‐rich diet in pregnancy. Healthcare access and the quality of antenatal care differ substantially, which is considered one of the main contributors. Al‐Kharabsheh et al. [13] tied maternal anemia in southern Jordan to education, income, diet, and frequency of antenatal care visits, suggesting possible barriers to timely, consistent care that may also affect pregnant women’s adherence to supplements. Tayyem et al. [21] reported that although supplement use was widespread in Jordan, it was often started late or not matched to clinical need, unlike the more systematic supplementation programs in Gulf countries.

Moreover, this study reported a higher prevalence than in earlier Jordanian studies. For instance, Arabyat et al. [11] estimated the prevalence in ever‐married women at 37.3% across different severities (20% mild, 16.3% moderate, and 1% severe), while Abdo et al. [12] estimated it at 27.4% among pregnant women (65.5% mild cases). The increase from 27.4% to 37.3% in earlier reports and to 52.5% in the current investigation required cautious interpretation. The difference may reflect methodological variations in sampling (targeted population), changes in nutritional patterns and healthcare access, differences in the targeted gestational age, and potential improvements in recognition of the condition through routine antenatal screening.

The history of three or more abortions carried the strongest effect (OR = 6.33). This is biologically plausible. Repeated pregnancies deplete maternal iron stores, and short interpregnancy intervals allow limited time for replenishment. Alternative explanations should also be considered. Women with multiple abortions may harbor undetected gynecological or chronic conditions that independently elevate anemia risk or may be of lower socioeconomic status. Longitudinal studies are needed to disentangle the independent contribution of abortion history from these factors.

The protective association between regular supplement use and lower odds of maternal anemia (OR = 0.104) is consistent with the literature. Yet a high proportion of our participants reported regular supplement intake (63.6%), suggesting other challenges, including late initiation, irregular dosing, and lack of awareness of absorption inhibitors, which might hinder their effectiveness in later stages of pregnancy. Physiological hemodilution also peaks in the third trimester, when expansion of plasma volume exceeds red blood cell production, which may place pregnant women at higher risk of developing anemia. This reinforces the clinical need for repeat Hb screening in the third trimester, rather than relying solely on early‐pregnancy assessment.

The convergence of both machine learning models on the same three predictors, namely supplement use, abortion history, and gestational trimester, supports their potential use in maternal risk categorization in central Jordan during routine antenatal care. This concordance across methods serves as a robustness check rather than a claim of superior predictive performance. Women presenting with these high‐risk profiles can be identified early for closer monitoring and targeted counseling, supporting a proactive rather than reactive clinical approach.

The current study’s unique contribution lies in its comprehensive analytical approach. Earlier Jordanian work examined prevalence, knowledge, nutrition, or obstetric factors one at a time; in this work, we analyzed reproductive risk factors, nutritional behavior, and maternal knowledge together. We also applied two models, multivariable logistic regression and an MLP, which provided predictive power beyond that of earlier descriptive studies and helped identify high‐risk groups for targeted intervention.

Educational and awareness interventions have a pivotal role in reducing maternal anemia prevalence among women of reproductive age. Jordanian knowledge‐focused and intervention studies suggest that structured health education enhances pregnant women’s awareness and dietary practices and can contribute to better maternal anemia control [19, 20]. Despite a high level of recorded awareness (88.2%), a considerable portion of our population (32.8%) demonstrates inadequate knowledge of critical aspects. This disparity suggests that educational initiatives should be tailored to help translate knowledge into practical health behaviors. Also, the observed prevalence suggests that previous educational initiatives did not sufficiently address maternal anemia across different stages of pregnancy.

The MoH in Jordan might use these findings to enhance antenatal detection and targeted prevention of maternal anemia. Given the high estimated prevalence of 52.5% and the higher risk observed in later stages of pregnancy, national antenatal care guidelines could recommend Hb screening at the first antenatal visit with repeat testing in the third trimester. These procedures might also be reinforced by clear referral and management pathways for cases of detected moderate/severe maternal anemia. MoH programs could also adopt a risk‐stratified response by identifying women with the strongest risk signals reported in this work (e.g., later gestation and adverse obstetric history) for closer follow‐up. This may help ensure that asymptomatic patients receive earlier assessment, appropriate counseling, and proactive or targeted follow‐up rather than waiting for the symptomatic presentation.

The results also show that many of these gaps can be targeted and eliminated early. Iron and folic acid should be provided to women early, ideally when pregnancy is confirmed or at least at the first antenatal visit, but simply handing out tablets is not enough. Women also need support to keep taking them, such as quick check‐ins, advice on managing side effects, and simple reminders. Alongside this, short, culturally appropriate talks on diet and anemia during routine visits could improve how much iron pregnant women take in and how consistently they take their supplements.

### 4.1. Implications for Nursing Practice and Education

Nurses are central to managing maternal anemia, especially early in pregnancy. Regular screening and nutrition counseling built into each woman’s care plan could help reduce prevalence. Nursing programs should also teach evidence‐based anemia prevention and care. And since these women come from different backgrounds, nurses need training in cross‐cultural communication.

### 4.2. Implications for Nursing Administration and Research

Health authorities should reach a consensus on a single standard protocol for screening and managing anemia at every stage of pregnancy. Research can go further, too. Machine‐learning tools that flag anemia risk are worth testing, as are the psychosocial factors behind what women eat and whether they stay on their supplements. Work in these areas would help shape better ways to reduce anemia.

### 4.3. Limitations

This study has some limitations. We applied a single WHO cutoff of 11.0 g/dL rather than a lower second‐trimester threshold. Because normal midpregnancy hemodilution can push Hb down without true iron deficiency, some second‐trimester women near the cutoff may have been classified as anemic, which could modestly inflate the overall estimate. It was also a cross‐sectional study that relied on self‐reported information, so some answers may not be fully accurate, even though nurses helped the women complete the questionnaire. In addition, we did not measure blood markers such as ferritin or signs of infection, so we could not identify the exact type of anemia. Finally, because the sample was fairly small (*n* = 297), the neural network results should be viewed with some caution, as models like this usually need larger datasets to train reliably. For this reason, the neural network was not treated as the main analysis but was used only as an additional check to see whether it identified the same key factors as logistic regression.

## 5. Conclusion

This study provides a broad assessment of maternal anemia in central Jordan by concurrently analyzing obstetric, nutritional, and knowledge‐related factors, thus surpassing the limitations of previous studies that considered only one factor. Maternal anemia affected 52.5% of the study participants, with some severe cases, indicating a considerable public health burden. It was most prevalent in women in the third trimester and among those with three or more previous abortions, and it was significantly associated with the absence of supplement use.

These findings, considering the known associations between maternal anemia and adverse outcomes such as postpartum hemorrhage, preterm birth, and low birth weight, point to the importance of integrated interventions in antenatal care, especially enhanced Hb screening in the third trimester, routine iron–folic acid supplementation with planned adherence support, and targeted education for women at high risk. The MoH could begin with screening. Hemoglobin should be checked at the first antenatal visit and again later in pregnancy, with clear routes for referral and treatment when it is low. Supplementation matters as much: iron and folic acid given early and taken consistently, with support to help women stay on them. Short nutrition and anemia sessions, suited to local habits and aimed at the women most at risk, would add to this. Delivered through routine services, none of this is out of reach, and together, it could lower the risk of anemia for mothers and babies across Jordan.

## Author Contributions

Noor Abu Eid, conceptualization, methodology, and data collection. Omar Abueed, conceptualization, formal analysis, software (machine‐learning modeling), writing–review and editing of the original draft, and project administration. Maya Abuarayes, formal analysis, validation, and review and editing. Reham Khresheh, supervision, conceptualization, methodology, writing–review and editing. Omar Abueed had full access to all of the data in this study and takes complete responsibility for the integrity of the data and the accuracy of the data analysis.

## Funding

The authors declare that the study received no funding.

## Disclosure

All authors have read and approved the final version of the manuscript.

## Ethics Statement

Ethical approvals were obtained from the Ministry of Health ethics committee (No. 11935, 2023 and No. 10513, 2023). The researcher explained the study’s purpose, participants’ rights, and the confidentiality of the information collected. Each participant signed an informed consent form, and we ensured that their participation was voluntary and anonymous. Participants were also informed that their involvement would not negatively affect their medical care, and they could withdraw from the study at any time. This study was conducted in accordance with the Declaration of Helsinki. Permission to use and adapt the study questionnaire was obtained from the corresponding author of the original instrument (Yousef M. Alflah) prior to data collection.

## Conflicts of Interest

The authors declare no conflicts of interest.

## Data Availability

The data that support the findings of this study are available from the corresponding author upon reasonable request.
